# Sex and race contribute to variation in mitochondrial function and insulin sensitivity

**DOI:** 10.14814/phy2.15049

**Published:** 2021-10-04

**Authors:** Gordon Fisher, Jeannie Tay, Jonathan L. Warren, W. Timothy Garvey, Ceren Yarar‐Fisher, Barbara A. Gower

**Affiliations:** ^1^ Departments of Human Studies University of Alabama at Birmingham Birmingham Alabama USA; ^2^ Departments of Nutrition Sciences University of Alabama at Birmingham Birmingham Alabama USA; ^3^ Singapore Institute of Clinical Sciences (SICS) Agency for Science, Technology and Research (A‐STAR) Singapore Singapore; ^4^ Departments of Medicine University of Alabama at Birmingham Birmingham Alabama USA

**Keywords:** insulin sensitivity, mitochondrial function, race, reactive oxygen species, sex

## Abstract

**Objective:**

Insulin sensitivity is lower in African American (AA) versus Caucasian American (CA). We tested the hypothesis that lower insulin sensitivity in AA could be explained by mitochondrial respiratory rates, coupling efficiency, myofiber composition, or H_2_O_2_ emission. A secondary aim was to determine whether sex affected the results.

**Methods:**

AA and CA men and women, 19–45 years, BMI 17–43 kg m^2^, were assessed for insulin sensitivity (SI_Clamp_) using a euglycemic clamp at 120 mU/m^2^/min, muscle mitochondrial function using high‐resolution respirometry, H_2_O_2_ emission using amplex red, and % myofiber composition.

**Results:**

SI_Clamp_ was greater in CA (*p *< 0.01) and women (*p *< 0.01). Proportion of type I myofibers was lower in AA (*p *< 0.01). Mitochondrial respiratory rates, coupling efficiency, and H_2_O_2_ production did not differ with race. Mitochondrial function was positively associated with insulin sensitivity in women but not men. Statistical adjustment for mitochondrial function, H_2_O_2_ production, or fiber composition did not eliminate the race difference in SI_Clamp_.

**Conclusion:**

Neither mitochondrial respiratory rates, coupling efficiency, myofiber composition, nor mitochondrial reactive oxygen species production explained lower SI_Clamp_ in AA compared to CA. The source of lower insulin sensitivity in AA may be due to other aspects of skeletal muscle that have yet to be identified.

## Graphical abstract

Present results do not support a role for mitochondrial function or fiber type in determining lower insulin sensitivity in African Americans. The source of lower skeletal‐muscle insulin sensitivity in African Americans remains unclear. Future research could focus on aspects of skeletal muscle composition, such as lipid species, and their association with insulin sensitivity in African Americans.

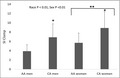

## 1 INTRODUCTION

Diabetes is the seventh leading cause of death in the United States and is expected to impact over 350 million people worldwide by 2030 (Wild et al., 2004). African Americans (AA) have consistently been shown to have higher rates of type 2 diabetes, lower insulin sensitivity, higher risk of diabetes complications, and higher overall mortality rates compared to Caucasian American (CA) counterparts (Beebe‐Dimmer et al., 2019; Hasson et al., 2015). To date, common risk factors such as obesity, visceral fat, socioeconomic status, and blood lipids have not been able to explain these differences in type 2 diabetes (T2D) risk (Albu et al., 1997; Egede et al., 2010; Hasson et al., 2015; O'Brien et al., 1989). However, lower insulin sensitivity in AA women can be statistically explained by skeletal muscle volume and lipid infiltration (Albu et al., 2005), suggesting that some aspect of muscle quality or composition is responsible. One possible component of skeletal muscle that may contribute to this observation is mitochondrial function.

Mitochondrial dysfunction has been linked to lower insulin sensitivity (Befroy et al., 2007; Lowell & Shulman, 2005). Recent evidence has shown potential differences in mitochondrial biology between AA and CA men and women. For example, DeLany et al. (2014) found lower mitochondrial oxidative capacity in nonobese AA women compared to nonobese CA women, and that lower insulin sensitivity was associated with reduced mitochondrial oxidative capacity in the entire cohort. While it remains to be determined if mitochondrial phenotypic differences can explain the racial disparity for T2D risk, a number of other physiological observations associated with mitochondrial function have been widely reported in the literature. For example, it has been shown that AA women have lower resting energy expenditure (Albu et al., 1997; Jakicic & Wing, 1998), reduced skeletal muscle fatty acid oxidation (Privette et al., 2003), lower maximal aerobic capacity (Hunter et al., 2000), and are more efficient during aerobic exercise compared to CA counterparts (Hunter et al., 2011; McCarthy et al., 2006). Reasons for these differences have been linked to differences in mitochondrial respiration, uncoupling protein 3 (UCP3) gene expression, and skeletal muscle myofiber composition (Ama et al., 1986; DeLany et al., 2014; Kimm et al., 2002; Tanner et al., 2002; Toledo et al., 2018). Therefore, it is plausible that ethnic differences in insulin sensitivity between AA and CA may be explained at least in part by differences in skeletal muscle mitochondrial function, myofiber composition, or downstream consequences of mitochondrial dysfunction.

To date, studies that have assessed the potential role of mitochondrial dysfunction and T2D risk have only included cohorts of women (DeLany et al., 2014; Kimm et al., 2002; Toledo et al., 2018). Whether these differences are sex‐specific remains to be determined. This is an important question given that differences in metabolism, fuel utilization, and insulin sensitivity have often been observed between men and women. For example, women have consistently been shown to have higher fatty acid oxidation rates during exercise compared to men (Lundsgaard & Kiens, 2014), and greater mitochondrial density in some (Montero et al., 2018) but not all studies (Tarnopolsky et al., 2007). Furthermore, it has consistently been shown in the literature that insulin resistance is more prevalent in men compared to premenopausal women (Lundsgaard & Kiens, 2014; Munguía‐Miranda et al., 2009) and that women appear to have higher rates of skeletal muscle glucose uptake relative to men (Kuhl et al., 2005; Paula et al., 1990). Thus, it is important to consider potential sex differences when trying to identify factors that explain the racial disparities in insulin sensitivity.

The primary objective of this study was to test the hypothesis that clamp‐derived, skeletal muscle‐specific, and insulin sensitivity (SI_Clamp_) would be significantly lower in AA compared to CA men and women, and that lower SI_Clamp_ in AA could be explained by differences in mitochondrial respiratory rates, coupling efficiency, skeletal muscle myofiber composition, or mitochondrial reactive oxygen species production (H_2_O_2_ emission). A secondary aim was to determine whether the results were consistent in men and women by testing relevant interaction terms.

## 2 METHODS

### 2.1 Study design, setting, and participants

This cross‐sectional study was conducted at the University of Alabama at Birmingham (UAB), between 2013 and 2018. One hundred and thirteen lean, overweight and obese AA and CA men and women, aged 19–45 years, who were sedentary to moderately active (<2 h/week of moderate, intentional, exercise) were recruited by public advertisement. A subset of 42 individuals in which all skeletal muscle, endocrine, body composition, and physiological assessments were completed and included for analysis in this manuscript. Individuals with diabetes were excluded from participation following a screening 75 g oral‐glucose‐tolerance test (2 h glucose ≥200 mg/dl). Other exclusion criteria included absence of regular menstrual cycle; pregnant, lactating or postmenopausal; smoking; not weight stable (change in weight >5 lbs.) in the previous 6 months; active engagement in unusual dietary practices (e.g., low‐carbohydrate diets); taking oral contraceptives; use of any medication known to affect carbohydrate or lipid metabolism, or energy expenditure; and use of antihypertensive agents that affect glucose tolerance (e.g., thiazide diuretics at doses >25 mg/days, angiotensin‐converting‐enzyme inhibitors). Participants were instructed to maintain their usual activity level, to avoid strenuous physical activity the day prior to testing, and to avoid all physical activity on the morning of testing. Women were tested 3–7 days after cessation of menstruation, while in the follicular phase of the menstrual cycle. All study assessments were conducted at the core facilities of the Center for Clinical and Translational Science (CCTS), Nutrition Obesity Research Center (NORC), and Diabetes Research Center (DRC). The UAB Institutional Review Board approved the study and all participants provided written informed consent.

### 2.2 Body composition

Body composition was assessed during the screening visit by total body scan from dual‐energy X‐ray absorptiometry (DXA) using a GE Lunar iDXA densitometer and Core Scan software (encore 15 [SPT]) GE Lunar Corporation. Participants were scanned in light clothing lying supine with arm at their sides. The scans were assessed for % fat, total body fat mass, and total lean mass.

### 2.3 Insulin sensitivity assessed by the hyperinsulinemic‐euglycemic glucose clamp (SI_Clamp_)

Skeletal muscle insulin sensitivity normalized to lean mass (SI_Clamp_) was assessed using the hyperinsulinemic‐euglycemic clamp. All SI_Clamp_ tests were performed outpatient in the Clinical Research Unit (CRU) at UAB’s CCTS after a ≥10 h overnight fast. With the participant in a recumbent position, an intravenous catheter was placed in an antecubital vein for insulin and glucose infusion. The insulin solution (regular Humulin, Eli Lilly & Co.) was prepared with normal saline and infused at 120 mU/m^2^/min for 3 h using an Alaris PC unit with Guardrails software (Carefusion Corp.). This dose of insulin, in a healthy, nondiabetic population, ensures that the glucose infusion rate reflects primarily skeletal muscle glucose uptake. Infusion of 20% dextrose was adjusted to maintain the blood glucose concentration at the individual's fasting level. Another catheter was placed in the contralateral arm for bedside measurement of blood glucose every 5‐min using a glucose analyzer (YSI 2300 STAT Plus, YSI, Inc.), and for blood sampling every 10‐min for later determination of serum glucose and insulin concentrations. The steady state period for each individual was defined as a ≥30‐min period that occurred ≥1 h after initiation of the insulin infusion, during which the coefficients of variation for blood glucose, serum insulin, and glucose infusion rate were less than 5%. Sera were analyzed for glucose (SIRRUS analyzer, Stanbio Laboratories) and insulin (TOSOH AIA‐II immunoassay analyzer, TOSCH Corp.) concentrations in the Core Laboratory of UAB’s Diabetes Research Center. The intra‐ and inter‐assay coefficients of variation for serum glucose and insulin concentrations were 1.28% and 2.48%, and 1.49% and 3.95%, respectively.

SI_Clamp_ (10^−4^ dl. kg^−1^ min^−1^/(μU/ml)) was defined as *M*/(*G* x Δ*I*), where *M* is the steady state glucose infusion rate (mg/kg lean mass/min), *G* is the steady state serum glucose concentration (mg/dl), and Δ*I* is the difference between basal and steady state serum insulin concentrations (μU/ml).

### 2.4 Muscle biopsies

Muscle biopsies were collected from a sub‐group of participants (*n* = 42). Skeletal muscle samples were obtained from the vastus lateralis under local anesthesia (1% lidocaine) using a 5‐mm Bergstrom biopsy needle with suction. The tissue was cleaned of adipose and connective tissue. A bundle of approximately 20 mg was selected for mitochondrial experiments and transported in Buffer X (50 mM K‐MES, 7.23 mM K_2_EGTA, 2.77 mM CaK_2_EGTA, 20 mM imidazole, 0.5 mM dithiothreitol, 20 mM taurine, 5.7 mM ATP, 14.3 mM phosphocreatine, and 6.56 mM MgCl_2_ [pH 7.1, 290 mOsm]) on ice (Perry et al., 2011). Portions used for immunohistochemistry were mounted cross‐sectionally on cork in optimum cutting temperature mounting medium mixed with tragacanth gum, frozen in liquid nitrogen‐cooled isopentane, and stored at −80°C.

### 2.5 Preparation of permeabilized muscle fiber bundles

This technique has been adapted from previously published methods (Warren et al., 2017). The biopsied tissue was dissected into several smaller muscle bundles (of approximately 1.0–5.0 mg wet weight). Each bundle was gently separated longitudinally with a pair of antimagnetic needle‐tipped forceps under magnification in Buffer X. Bundles were weighed and then treated with 30 µg/ml saponin in Buffer X on a rotator for 30 min at 4℃. Next, the tissue bundles were washed for 15 min in Buffer Z (105 mM K‐MES, 30 mM KCl, 1 mM EGTA, 10 mM K_2_HPO_4_, 5 mM MgCl_2_, 5 µM glutamate, 2 µM malate, and 5.0 mg/ml BSA (pH 7.4, 290 mOsm)). Finally, the samples were transferred to Buffer Z supplemented with 5 µM blebbistatin and 20 mM creatine hydrate for 10 min prior to experiments. Blebbistatin was present during all respirometry and H_2_O_2_ experiments to prevent contraction of the myofibers (Perry et al., 2011).

### 2.6 Mitochondrial respiration

High‐resolution respirometry experiments were performed using an Oxygraph O2K (Oroboros Instruments, Innsbruck, Austria) containing 2 ml of Buffer Z with 5 µM blebbistatin and 20 mM creatine hydrate, constantly stirred at 37℃ under conditions of O_2_ saturation, containing a prepared permeabilized fiber bundle of approximately 3.0–5.0 mg. Two substrate protocols were used: 9 mM pyruvate, 4 mM malate, and 2.5 mM succinate (PMS) to drive convergent electron input to complexes I and II of the ETS or 40 μM palmitoyl carnitine and 2 mM malate (PCM) to examine mitochondrial fatty acid oxidation. State 3 in each substrate condition was measured after the addition of 1 mM ADP. Cytochrome *c* (10 μM) was added to assess the mitochondrial membrane integrity. There was no significant difference in State 3 respiration assessed after cytochrome *c* addition. State 4 was induced by the addition of 2 μg/ml oligomycin. Oxygen flux was normalized to the wet weight of each fiber bundle taken prior to experiments (Pesta & Gnaiger, 2012). The respiratory control ratio (RCR) was calculated as State 3/State 4, providing a measure of mitochondrial coupling.

### 2.7 H_2_O_2_ quantification

Using a permeabilized fiber bundle of approximately 1.0–1.5 mg, H_2_O_2_ emission was measured fluorometrically at 37℃ in Buffer Z containing 5 µM blebbistatin, 20 mM creatine hydrate, 10 µM Amplex Ultra Red, and 3 U/ml horseradish peroxidase. Oxidation of Amplex Ultra Red to resorufin was monitored using a Fluoromate SF‐2 spectrofluorometer (SCINCO, Seoul, South Korea) with temperature control and magnetic stirring at more than 1000 rpm detecting at excitation/emission *λ* = 568/581 nm. For each experiment, resorufin fluorescence was converted to pmol H_2_O_2_ via an H_2_O_2_ standard curve generated under identical substrate conditions with the exception of the permeabilized fiber bundles. H_2_O_2_ emission during coupled respiration (State 3) was measured after the addition of 9 mM pyruvate, 4 mM malate, 2.5 mM succinate, and 1 mM ADP. H_2_O_2_ emission during uncoupled respiration (State 4) was measured after the addition of 2 μg/ml oligomycin. H_2_O_2_ emission was normalized to the wet weight of the fiber bundle taken prior to each experiment. Not all muscle samples yielded measurable H_2_O_2_; thus data are reported for only those samples with measurable data (*n* = 30).

### 2.8 Myofiber type distribution

All visible connective and adipose tissues were removed from the biopsy samples with the aid of a dissecting microscope. Portions used for immunohistochemistry were mounted cross‐sectionally on cork in optimum cutting temperature mounting medium mixed with tragacanth gum, frozen in liquid nitrogen‐cooled isopentane, and stored at −80℃. The relative distribution of myofiber types I, IIa, and IIx was determined by myosin heavy chain immunohistochemistry using our well‐established protocol (Kim et al., 2005).

### 2.9 VO_2_ during submaximal treadmill exercise

During a separate test day, participants came to the laboratory and performed a treadmill walking test following an overnight fast. This test consisted of walking on a treadmill for 4 min at 2.5 and 3 mph submaximal VO_2_ and heart rate were obtained during steady state exercise in the third and fourth minute for each speed. These data were obtained to compare economy at the whole‐body level with coupling efficiency within skeletal muscle mitochondria.

### 2.10 Statistical analyses

All statistical analyses were conducted using SPSS Statistics for Macintosh Version 22.0 (IBM Corp.). Descriptive statistics and primary outcome variables are reported by race and gender as mean ± standard deviation. A univariate ANOVA was used to compare race, sex, and race x sex interactions for SI_Clamp_, body composition, blood lipids, blood pressure, mitochondrial respiratory rates, H_2_O_2_ emission, and myofiber composition. When significant interactions were observed, Bonferroni pairwise comparisons were used to compare differences between race and sex. Pearson correlation coefficients were used to examine associations among variables. Correlations were examined in all participants combined, and by sex or race when interactions were observed. A one‐way ANCOVA was performed to determine if observed race and sex differences in SI_Clamp_ persisted when controlling for differences in H_2_O_2_ emission. Because % type I myofiber differed with race, this variable was also included in the final ANCOVA.

## 3 RESULTS

### 3.1 Anthropometric and metabolic measurements

Descriptive characteristics of the study population are presented in Table 1 by race and sex. There were no differences in age between AA and CA or between men and women. AA had significantly greater lean mass and systolic blood pressure compared to CA (*p *< 0.01), whereas SI_Clamp_ was significantly lower in AA compared to CA (*p *< 0.01). Women had significantly greater %body fat, SI_Clamp_, and HDL‐C compared to men (*p *< 0.05), whereas lean mass, and systolic blood pressure were significantly lower in women compared to men (*p *< 0.05).

**TABLE 1:** Main effect and interactions between race and sex on age, body composition, insulin sensitivity, glycaemia, and other cardiovascular disease risk factors

	Men (*n* =18)		Women (*n* = 24)		Race	Sex	Race*Sex
	CA (*n* = 8)	AA (*n* = 10)	CA (*n* = 9)	AA (*n* = 15)	*p*	*p*	*p*
Age (years)	30 (9)	28 (9)	28 (7)	31 (8)	0.477	0.892	0.531
BMI (kg m^2^)	28 (5)	25 (3)	25 (3)	25 (2)	0.217	0.955	0.804
Fat mass (kg)	20 (8)	29 (12)	27 (9)	29 (12)	0.24	0.181	0.545
Body fat (%)	26 (8)	27 (11)	36 (8)	36 (8)	0.891	**<0.001**	0.705
Lean mass (kg)	55 (7)	63 (8)	42 (3)	45 (5)	**<0.01**	**<0.001**	0.137
SI _Clamp_ lean mass (10^−4^ kg min^−1^/(μU/ml)	6.9 (2.9)	3.9 (1.5)	9.0 (3.4)	5.7 (2.2)	**<0.003**	**<0.001**	0.541
Fasting glucose (mg/dl)	85.4 (8.6)	92.2 (12.9)	88.8 (9.9)	87.8 (5.5)	0.502	0.839	0.181
Fasting insulin (μU/ml)	5.7 (3.4)	13.9 (21.4)	6.9 (2.9)	7.6 (2.4)	0.442	0.455	0.281
Total cholesterol (mg/dl)	164.9 (31.7)	167.6 (32.5)	160.8 (27.0)	177.5 (25.1)	0.524	0.744	0.439
Triglycerides (mg/dl)	90.6 (39.2)	112.2 (125.1)	72.3 (21.6)	52.9 (13.3)	0.999	0.067	0.327
HDL (mg/d)	55.1 (8.9)	52.3 (7.2)	62.7 (12.0)	63.2 (8.1)	0.686	**0.002**	0.542
LDL (mg/d)	91.6 (25.3)	91.4 (15.4)	83.6 (24.4)	103.7 (23.1)	0.157	0.753	0.147
SBP (mmHg)	116 (13)	125 (10)	108 (4)	117 (13)	**0.012**	**0.016**	0.878
DBP (mmHg)	67 (8)	70 (9)	66 (8)	68 (10)	0.25	0.46	0.85

Abbreviations: AA, African‐Americans; DBP, Diastolic Blood Pressure; EA, Caucasian‐Americans; HDL, High‐density lipoprotein cholesterol; LDL, Low‐density lipoprotein cholesterol; SBP, Systolic Blood Pressure; SI_Clamp,_ Lean mass, skeletal muscle insulin sensitivity assessed by the hyperinsulinemic‐euglycemic glucose clamp and adjusted for total lean mass.

Analyses by univariate ANOVA. Data are unadjusted means (SD).

**FIGURE 1:**
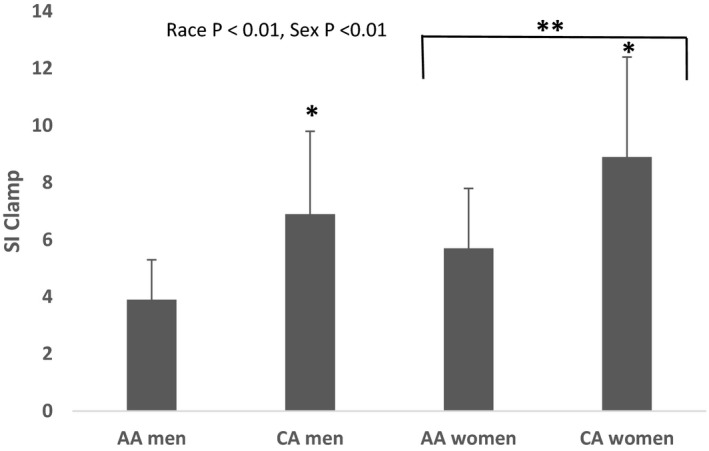
Differences in insulin sensitivity based on race and sex. Main effects of race (*p *< 0.01) and sex (*p *< 0.01) were observed. No race *x* sex interactions were observed. SI _clamp_ was significantly greater in CA compared to AA (*p *< 0.01), and SI _clamp_ was significantly greater in women compared to men (*p* < 0.01) (*N* = 30)

Insulin sensitivity data from the hyperinsulinemic‐euglycemic clamp based on race, sex, and race*sex are presented in Figure 1. There were significant main effects of sex and race, with insulin sensitivity being greater in women (*p *< 0.01) and CA (*p *< 0.01). No race*sex interaction was observed.

### 3.2 Skeletal muscle, mitochondrial respirometry, and H_2_O_2_ production

**FIGURE 2:**
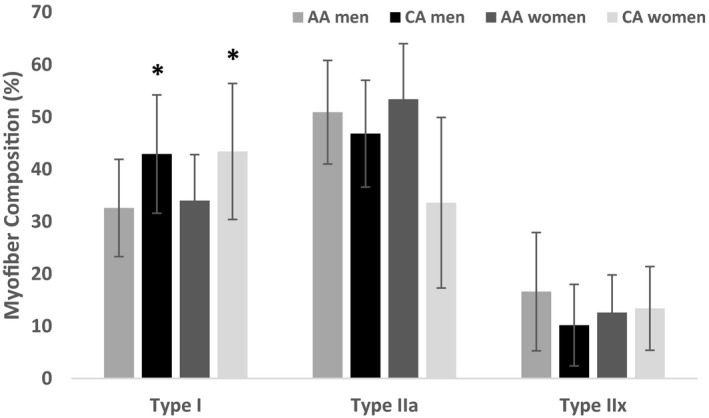
Differences in skeletal muscle myofiber composition based on race and sex. Type I myofiber composition was significantly greater in CA men and women compared to AA men and women (*p *< 0.01). No significant sex differences between Type I myofiber composition was observed. No significant interactions for race or sex were observed for Type IIa or IIX myofiber composition

**FIGURE 3:**
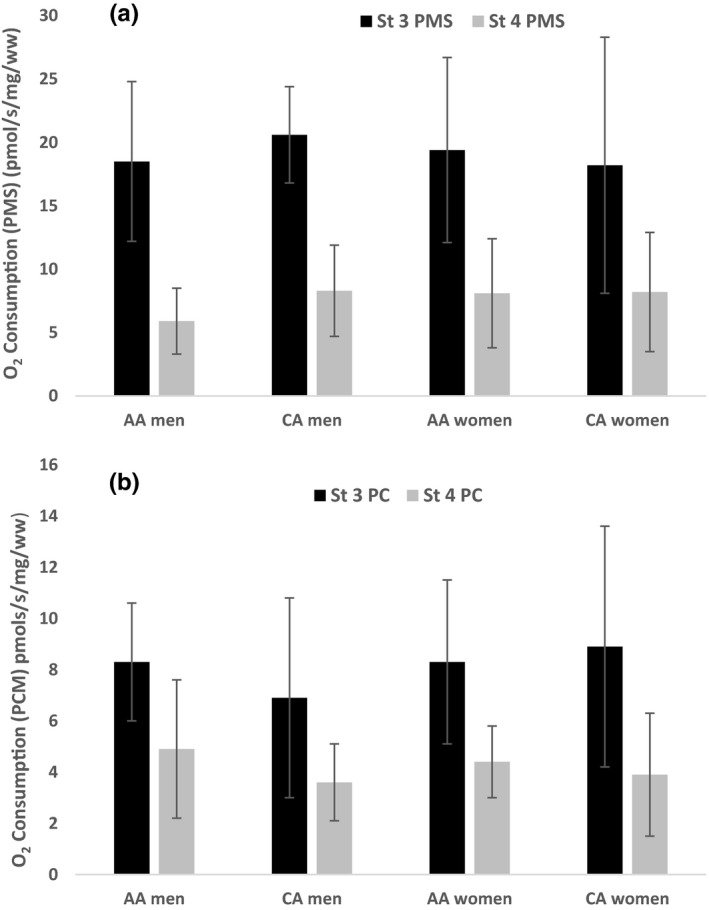
Differences in skeletal muscle mitochondrial respiration based on race and sex. (a) State 3 and State 4 respiration supported by pyruvate, malate and succinate (PMS); (b) State 3 and State 4 respiration supported by malate and palmitoyl carnitine (PCM). No significant race, sex, or race x sex interactions were observed for mitochondrial respiratory rates

**FIGURE 4:**
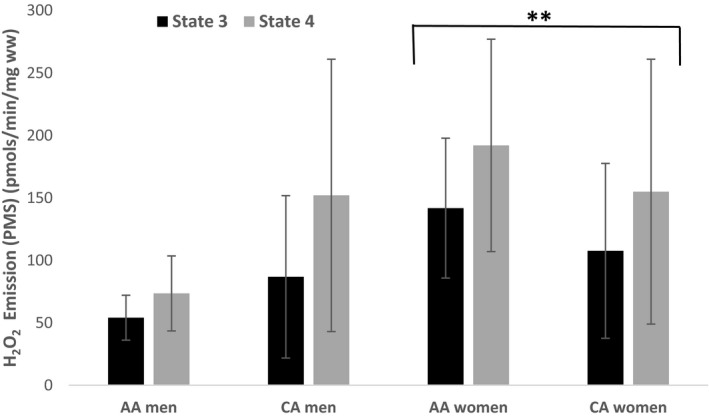
Differences in skeletal muscle mitochondrial H2O2 production based on race and sex. H_2_O_2_ emission during State 3 and State 4 respiration supported by malate, pyruvate, and succinate was significantly higher in women compared to men **(*p* < 0.05). No race differences were observed

Skeletal muscle myofiber composition is presented in Figure 2 by race and sex. A significant main effect of race was observed such that Type I myofiber composition was significantly greater in CA compared to AA (*p *< 0.01). No significant sex differences were observed. Additionally, there were no significant race or sex interactions observed for either type IIa or IIx myofiber composition. Mitochondrial State 3 and State 4 respiratory rates are shown in Figure 3a,b. No significant differences between race or sex were observed for State 3‐ (*p *= 0.426; *p *= 0.985), State 4‐respiration (*p* = 0.504; *p* = 0.360) or the RCR (*p* = 0.493; *p* = 0.886) supported by PMS or PC. Skeletal muscle ROS production for the sub‐sample is presented in Figure 4. H_2_O_2_ emission during State 3 and State 4 respiration supported by malate, pyruvate, and succinate were significantly higher in women compared to men (*p* < 0.05). The main effect of race was not significant.

### 3.3 VO_2_ responses during treadmill walking

**FIGURE 5:**
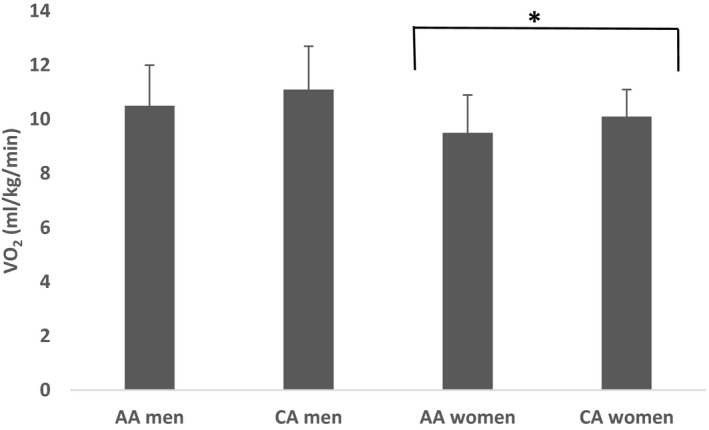
Differences in skeletal muscle mitochondrial H_2_O_2_ production based on race and sex. H_2_O_2_ emission during State 3 and State 4 respiration supported by malate, pyruvate, and succinate was significantly higher in women compared to men (*p *< 0.05). No race differences were observed

The VO_2_ responses are shown in Figure 5. There were significant sex effects for VO_2_ during 2.5 mph treadmill walking (*p *< 0.05). VO_2_ was significantly lower in women compared to men. (*p* < 0.05).

No main effects of race were observed. There were no significant associations between submaximal walking VO_2_ and mitochondrial respiratory rates.

### 3.4 Associations between insulin sensitivity, skeletal muscle, mitochondria, and ROS

**FIGURE 6:**
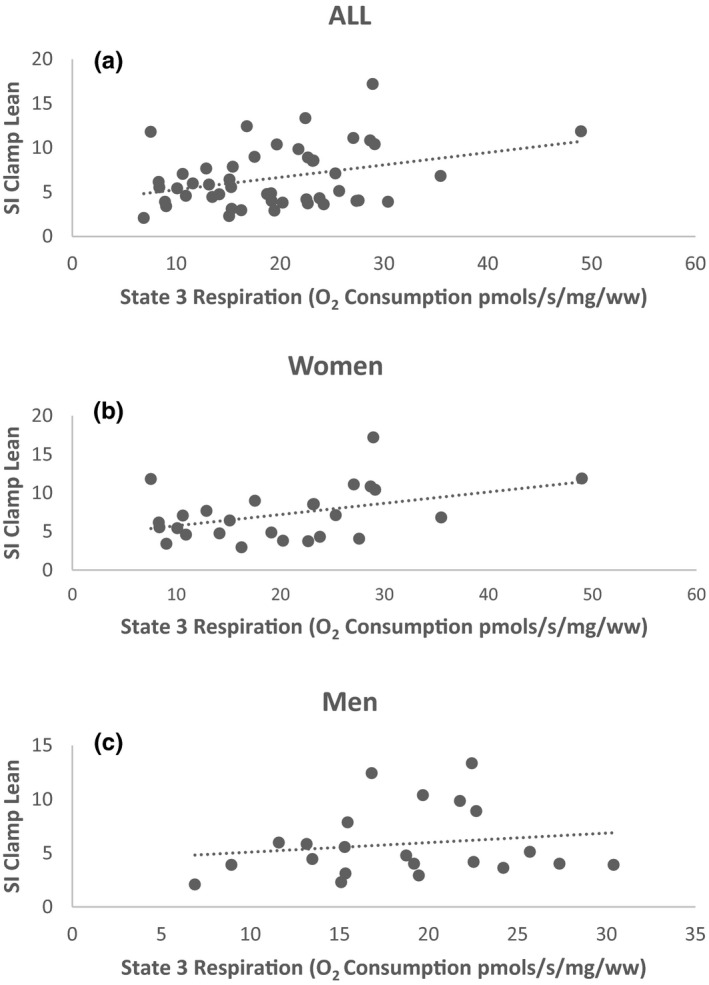
Mitochondrial State 3 respiration (PMS) was positively correlated with SIClampLean mass in the entire cohort (*r*=0.335, *p* < 0.05) (a). However when dichotomized based on sex we found that this correlation only occurred in women (*r*=0.393, *p* < 0.05) (b) not men (*r*=0.171, *p* =0.447) (c)

Mitochondrial state 3 respiration was positively associated with SI_Clamp_ in the entire cohort (*r* = 0.335, *p* < 0.05). (Figure 6a). However, when we dichotomized based on sex we found that this association occurred in women (*r *= 0.393, *p* < 0.05) (Figure 6b) but not men (*r* = 0.171, *p *= 0.447) (Figure 6c). Additionally, the RCR was positively associated with St 3 (*r* = 0.408, *p *< 0.05), St 4 (*r* = 0.471, *p* < 0.05), and rotenone (*r* = 0.532, *p* < 0.01) H_2_O_2_. No significant associations were observed between SI_Clamp,_ and myofiber type or H_2_O_2_ emission.

### 3.5 Insulin sensitivity ANCOVA analyses

There continued to be a significant effect of race on SI_Clamp_ after controlling for % type I myofiber composition, *F* (1, 36 = 15.79, *p *< 0.001). Additionally, given that H_2_O_2_ emission was higher in women compared to men, we performed a one‐way ANCOVA analyses dichotomized by sex to determine if there were differences in SI_Clamp_ within sex while controlling for H_2_O_2_ emission. Race differences in SI_Clamp_ in both men, *F* (1, 11 = 7.48, *p *< 0.05) and women, *F* (1, 17 = 4.744, *p *< 0.05) persisted after controlling for H_2_O_2_ emission.

## 4 DISCUSSION

The primary goal of this study was to determine if lower insulin sensitivity in AA would be explained by differences in mitochondrial function, skeletal muscle myofiber composition, or mitochondrial reactive oxygen species production (H_2_O_2_ emission). Although proportion of type I fibers was lower in AA, we found no race differences in skeletal muscle mitochondrial respiratory rates, coupling efficiency, or skeletal muscle H_2_O_2_ production. Further, inclusion of these variables in statistical models for insulin sensitivity did not alter the independent effect of race. Thus, present results do not support a role for mitochondrial function or fiber type in determining lower insulin sensitivity in AA.

To date, data are inconclusive regarding factors that may increase the risk of insulin resistance and T2D in AA compared to CA. Insulin resistance in AA compared to CA, at least in lean women, is specific to skeletal muscle; that is, race differences in hepatic insulin sensitivity are not observed (DeLany et al., 2014). Further, statistical adjustment for skeletal muscle volume and lipid infiltration explains lower whole‐body insulin sensitivity in AA versus CA women (Albu et al., 2005). It has been postulated that some aspect of muscle quality or composition in AA, such as lower mitochondrial oxidative capacity or lower type I fiber proportion, may explain these differences. Although a previous investigation demonstrated lower mitochondrial oxidative capacity in lean (normal‐weight) AA women compared to lean CA women (DeLany et al., 2014), we did not observe this race difference. While we can only speculate regarding the reason for the difference in results, it is important to point out that our study included men and women as well as lean and obese individuals, whereas the earlier study (DeLany et al., 2014) included only lean women. It is possible that differences in adiposity levels or sex may explain these discrepant findings. We have previously demonstrated that mitochondrial oxidative capacity is positively associated with adiposity (Fisher et al., 2017); thus it is possible that differences in the body composition of study participants contributed to the different results between studies.

Muscle fiber type distribution, specifically lower percentage of type I myofiber in AA, has been hypothesized to be a primary inherent factor in AA that predispose them to insulin resistance and T2D (Nielsen & Christensen, 2011). The rationale for this hypothesis is that the greater oxidation of lipid fuel by type I relative to type II fibers may limit accumulation of specific lipid species in the myocyte that could impair insulin signaling. Both extramyocellular lipid (Lawrence et al., 2011) and inter‐muscular adipose tissue (Gallagher et al., 2005) are higher in AA relative to CA. We (Fisher et al., 2017) and others (Kriketos et al., 1996; Stuart et al., 2013) have found a positive association between type I myofiber percent and insulin sensitivity, similar to the trend that was observed in the present study (*p *= 0.08). Additionally, we found a significantly lower type I myofiber percentage in AA compared to CA in this study; however this did not explain the race differences in insulin sensitivity. Therefore, although there were differences between type I myofiber composition between AA and CA, the observed racial disparity in insulin sensitivity does not appear to be due to these differences.

We also wanted to determine if sex affected associations between mitochondrial function and insulin sensitivity. While we did not observe sex differences in mitochondrial respiration between men and women, similar to an earlier study (DeLany et al., 2014), we found that insulin sensitivity was positively associated with mitochondrial respiratory rates in all women combined. In contrast, mitochondrial respiration was not associated with insulin sensitivity in men. While we cannot establish causality from these data, these observations suggest that insulin sensitivity may be more closely linked to mitochondrial function in women than in men. We also found greater skeletal muscle H_2_O_2_ production in women compared to men. Indeed, higher circulating markers of oxidative stress have been observed in women when compared to men (Brunelli et al., 2014). Results from the present study suggest that mitochondrial production of ROS may be inherently higher in women than men. However, given that women have higher insulin sensitivity than men, there may not be a direct connection between H_2_O_2_ production and insulin sensitivity. It is also possible that greater H_2_O_2_ production is associated with greater insulin sensitivity as a recent study in rodents found insulin resistance to be associated with lower H_2_O_2_ production (McMurray et al., 2019). Thus, further research is warranted to determine the link between ROS production and insulin sensitivity.

The role of mitochondrial function as a primary or secondary mediator of the onset of insulin resistance and T2D has received a great deal of attention during the past 20 years; however, the relationship between mitochondrial function and insulin resistance remains controversial. There are a number of studies that have shown impaired mitochondrial function concurrently with reduced insulin sensitivity (Petersen et al., 2003; Short et al., 2005), however there is also evidence that changes in insulin sensitivity and mitochondrial function are not associated (Lalia et al., 2016) and may occur independently of one another (Hancock et al., 2008; Toledo et al., 2008). Given the observed sex differences in the present study, it is possible that equivocal findings found in previous studies could be due in part to confounding sex differences in the association of mitochondrial function and insulin sensitivity.

Oxygen uptake during treadmill exercise has been shown to be lower in AA compared to CA in many previous investigations by our group (Hunter et al., 2011; McCarthy et al., 2006) and others (Weston et al., 2000), in which AA are more economical during exercise compared to CA counterparts. In this study there was an overall mean difference in submaximal oxygen uptake between AA and C men and women however these differences were not statistically significant. While there was not a significant difference in this subset, there was a statistically significant difference in the entire study cohort. We initially postulated that potential whole‐ body energetic differences would also be observed within skeletal muscle mitochondria. However, we did not see any differences in mitochondrial coupling efficiency or oxidative phosphorylation between AA and CA men and women. Additionally, there was no association between mitochondrial coupling efficiency and walking economy during exercise. The mitochondrial efficiency hypothesis postulates that genetic adaptations may occur when individuals are exposed to different global environments (Mishmar et al., 2003). For example, when individuals are exposed to hot tropical climates, it would be more beneficial to generate ATP while minimizing heat production, whereas cold climates would favor more uncoupled ATP production and greater heat production. This logic has been proposed as a potential reason that individuals with European origins may have greater uncoupling. y(Fridlyand & Philipson, 2006). However, a downside to having greater coupling efficiency may be excess ROS production, particularly under conditions of fuel excess. Our data support this idea as we found a positive correlation between the respiratory control ratio and skeletal muscle hydrogen peroxide production. To our knowledge, differences in skeletal muscle mitochondrial coupling efficiency between AA and CA have not been shown. Thus, it remains to be determined if energetic efficiency within the mitochondria differs with race, and if it plays a role in T2D risk.

Strengths of this study include the use of a relatively large sample of lean, overweight and obese AA and CA men and women. Additionally, we assessed insulin sensitivity using the reference standard euglycemic‐hyperinsulinemic clamp and assessed mitochondrial respiration using high‐resolution respirometry in permeabilized muscle fiber combined with the assessment of H_2_O_2_ production. A primary limitation in this study is the cross‐sectional nature of the study, which does not establish causality.

In conclusion, mitochondrial function was positively associated with insulin sensitivity in women but not men. Greater type I fiber percent in CA was not associated with insulin sensitivity or mitochondrial function. Neither mitochondrial respiratory rates, coupling efficiency, myofiber composition, nor mitochondrial reactive oxygen species production explained lower insulin sensitivity in AA compared to CA. The source of lower skeletal muscle insulin sensitivity in AA remains unclear. Future research could focus on aspects of skeletal muscle composition, such as lipid species, and their association with insulin sensitivity in AA.

## AUTHOR CONTRIBUTIONS

GF and BAG have full access to all data in the study and take responsibility for the integrity of the data and the accuracy of the data analysis. Study concept and design: All authors. Analysis and interpretation of data: GF, JT, and BAG. Drafting of the manuscript: GF and BAG. Critical revision the manuscript for intellectual content: All authors. Read and approved the final manuscript: All authors. Obtained funding: WTG, GF, and BAG.

## CLINICAL TRIALS REGISTRATION

Race Adiposity Interactions Regulate Mechanisms Determining Insulin Sensitivity NCT03043235 https://clinicaltrials.gov/ct2/show/NCT03043235.

## RESOURCE SHARING PLAN

The data collected from this study will be shared as part of the NIH data repository. The UAB IRB will receive a data‐sharing plan for this project to be evaluated prior to the initiation of the study and will be requested to verify that the submission and sharing of data, the informed consent from study participants, the de‐identification of datasets, the collection of the data, and the consideration of participant's risk is in accordance with the NIH Policy and procedures.

## References

[phy215049-bib-0001] Albu, J. B., Kovera, A. J., Allen, L., Wainwright, M., Berk, E., Raja‐Khan, N., Janumala, I., Burkey, B., Heshka, S., & Gallagher, D. (2005). Independent association of insulin resistance with larger amounts of intermuscular adipose tissue and a greater acute insulin response to glucose in African American than in white nondiabetic women. The American Journal of Clinical Nutrition, 82(6), 1210–1217. 10.1093/ajcn/82.6.1210 16332653 PMC2670467

[phy215049-bib-0002] Albu, J., Shur, M., Curi, M., Murphy, L., Heymsfield, S. B., & Pi‐Sunyer, F. X. (1997). Resting metabolic rate in obese, premenopausal black women. The American Journal of Clinical Nutrition, 66(3), 531–538. 10.1093/ajcn/66.3.531 9280169

[phy215049-bib-0003] Ama, P. F., Simoneau, J. A., Boulay, M. R., Serresse, O., Thériault, G., & Bouchard, C. (1986). Skeletal muscle characteristics in sedentary black and Caucasian males. Journal of Applied Physiology (1985), 61(5), 1758–1761. 10.1152/jappl.1986.61.5.1758 2946652

[phy215049-bib-0004] Beebe‐Dimmer, J. L., Ruterbusch, J. J., Cooney, K. A., Bolton, A., Schwartz, K., Schwartz, A. G., & Heath, E. (2019). Racial differences in patterns of treatment among men diagnosed with de novo advanced prostate cancer: A SEER‐Medicare investigation. Cancer Medicine, 8(6), 3325–3335. 10.1002/cam4.2092. 31094098 PMC6558501

[phy215049-bib-0005] Befroy, D. E., Petersen, K. F., Dufour, S., Mason, G. F., de Graaf, R. A., Rothman, D. L., & Shulman, G. I. (2007). Impaired mitochondrial substrate oxidation in muscle of insulin‐resistant offspring of type 2 diabetic patients. Diabetes, 56(5), 1376–1381. 10.2337/db06-0783 17287462 PMC2995532

[phy215049-bib-0006] Brunelli, E., Domanico, F., La Russa, D., & Pellegrino, D. (2014). Sex differences in oxidative stress biomarkers. Current Drug Targets, 15(8), 811–815. 10.2174/1389450115666140624112317. 24958098

[phy215049-bib-0007] DeLany, J. P., Dubé, J. J., Standley, R. A., Distefano, G., Goodpaster, B. H., Stefanovic‐Racic, M., Coen, P. M., & Toledo, F. G. S. (2014). Racial differences in peripheral insulin sensitivity and mitochondrial capacity in the absence of obesity. The Journal of Clinical Endocrinology & Metabolism, 99(11), 4307–4314. 10.1210/jc.2014-2512. 25105736 PMC4223429

[phy215049-bib-0008] Egede, L. E., Mueller, M., Echols, C. L., & Gebregziabher, M. (2010). Longitudinal differences in glycemic control by race/ethnicity among veterans with type 2 diabetes. Medical Care, 48(6), 527–533. 10.1097/MLR.0b013e3181d558dc. 20473215

[phy215049-bib-0009] Fisher, G., Windham, S. T., Griffin, P., Warren, J. L., Gower, B. A., & Hunter, G. R. (2017). Associations of human skeletal muscle fiber type and insulin sensitivity, blood lipids, and vascular hemodynamics in a cohort of premenopausal women. European Journal of Applied Physiology, 117(7), 1413–1422. 10.1007/s00421-017-3634-9. 28497385 PMC5963254

[phy215049-bib-0010] Fridlyand, L. E., & Philipson, L. H. (2006). Cold climate genes and the prevalence of type 2 diabetes mellitus. Medical Hypotheses, 67(5), 1034–1041. 10.1016/j.mehy.2006.04.057. 16797871

[phy215049-bib-0011] Gallagher, D., Kuznia, P., Heshka, S., Albu, J., Heymsfield, S. B., Goodpaster, B., Visser, M., & Harris, T. B. (2005). Adipose tissue in muscle: A novel depot similar in size to visceral adipose tissue. The American Journal of Clinical Nutrition, 81(4), 903–910. 10.1093/ajcn/81.4.903. 15817870 PMC1482784

[phy215049-bib-0012] Hancock, C. R., Han, D. H., Chen, M., Terada, S., Yasuda, T., Wright, D. C., & Holloszy, J. O. (2008). High‐fat diets cause insulin resistance despite an increase in muscle mitochondria. Proceedings of the National Academy of Sciences, 105(22), 7815–7820. 10.1073/pnas.0802057105. PMC2409421 18509063

[phy215049-bib-0013] Hasson, B. R., Apovian, C., & Istfan, N. (2015). Racial/ethnic differences in insulin resistance and beta cell function: Relationship to racial disparities in type 2 diabetes among African Americans versus Caucasians. Current Obesity Reports, 4(2), 241–249. 10.1007/s13679-015-0150-2. 26627219

[phy215049-bib-0014] Hunter, G. R., McCarthy, J. P., Bamman, M. M., Larson‐Meyer, D. E., Fisher, G., & Newcomer, B. R. (2011). Exercise economy in African American and European American women. European Journal of Applied Physiology, 111(8), 1863–1869. 10.1007/s00421-010-1816-9. 21229260 PMC3137679

[phy215049-bib-0015] Hunter, G. R., Weinsier, R. L., Darnell, B. E., Zuckerman, P. A., & Goran, M. I. (2000). Racial differences in energy expenditure and aerobic fitness in premenopausal women. The American Journal of Clinical Nutrition, 71(2), 500–506. 10.1093/ajcn/71.2.500. 10648264

[phy215049-bib-0016] Jakicic, J. M., & Wing, R. R. (1998). Differences in resting energy expenditure in African‐American vs Caucasian overweight females. International Journal of Obesity, 22(3), 236–242. 10.1038/sj.ijo.0800575 9539192

[phy215049-bib-0017] Kim, J. S., Kosek, D. J., Petrella, J. K., Cross, J. M., & Bamman, M. M. (2005). Resting and load‐induced levels of myogenic gene transcripts differ between older adults with demonstrable sarcopenia and young men and women. Journal of Applied Physiology (1985), 99(6), 2149–2158. 10.1152/japplphysiol.00513.2005 16051712

[phy215049-bib-0018] Kimm, S. Y., Glynn, N. W., Aston, C. E., Damcott, C. M., Poehlman, E. T., Daniels, S. R., & Ferrell, R. E. (2002). Racial differences in the relation between uncoupling protein genes and resting energy expenditure. The American Journal of Clinical Nutrition, 75(4), 714–719. 10.1093/ajcn/75.4.714 11916758

[phy215049-bib-0019] Kriketos, A. D., Pan, D. A., Lillioja, S., Cooney, G. J., Baur, L. A., Milner, M. R., Sutton, J. R., Jenkins, A. B., Bogardus, C., & Storlien, L. H. (1996). Interrelationships between muscle morphology, insulin action, and adiposity. American Journal of Physiology‐Regulatory, Integrative and Comparative Physiology, 270(6 Pt 2), R1332–R1339. 10.1152/ajpregu.1996.270.6.R1332 8764301

[phy215049-bib-0020] Kuhl, J., Hilding, A., Ostenson, C. G., Grill, V., Efendic, S., & Båvenholm, P. (2005). Characterisation of subjects with early abnormalities of glucose tolerance in the Stockholm Diabetes Prevention Programme: The impact of sex and type 2 diabetes heredity. Diabetologia, 48(1), 35–40. 10.1007/s00125-004-1614-1 15619073

[phy215049-bib-0021] Lalia, A. Z., Dasari, S., Johnson, M. L., Robinson, M. M., Konopka, A. R., Distelmaier, K., Port, J. D., Glavin, M. T., Esponda, R. R., Nair, K. S., & Lanza, I. R. (2016). Predictors of whole‐body insulin sensitivity across ages and adiposity in adult humans. The Journal of Clinical Endocrinology & Metabolism, 101(2), 626–634. 10.1210/jc.2015-2892 26709968 PMC4880121

[phy215049-bib-0022] Lawrence, J. C., Newcomer, B. R., Buchthal, S. D., Sirikul, B., Oster, R. A., Hunter, G. R., & Gower, B. A. (2011). Relationship of intramyocellular lipid to insulin sensitivity may differ with ethnicity in healthy girls and women. Obesity, 19(1), 43–48. 10.1038/oby.2010.148 20559297 PMC3204213

[phy215049-bib-0023] Lowell, B. B., & Shulman, G. I. (2005). Mitochondrial dysfunction and type 2 diabetes. Science, 307(5708), 384–387. 10.1126/science.1104343 15662004

[phy215049-bib-0024] Lundsgaard, A. M., & Kiens, B. (2014). Gender differences in skeletal muscle substrate metabolism—Molecular mechanisms and insulin sensitivity. Frontiers in Endocrinology (Lausanne), 5, 195. 10.3389/fendo.2014.00195 PMC4230199 25431568

[phy215049-bib-0025] McCarthy, J. P., Hunter, G. R., Larson‐Meyer, D. E., Bamman, M. M., Landers, K. A., & Newcomer, B. R. (2006). Ethnic differences in triceps surae muscle‐tendon complex and walking economy. The Journal of Strength and Conditioning Research, 20(3), 511–518. 10.1519/17395.1 16937962

[phy215049-bib-0026] McMurray, F., MacFarlane, M., Kim, K., Patten, D. A., Wei‐LaPierre, L., Fullerton, M. D., & Harper, M.‐E. (2019). Maternal diet‐induced obesity alters muscle mitochondrial function in offspring without changing insulin sensitivity. The FASEB Journal, 33(12), 13515–13526. 10.1096/fj.201901150R 31581846

[phy215049-bib-0027] Mishmar, D., Ruiz‐Pesini, E., Golik, P., Macaulay, V., Clark, A. G., Hosseini, S., Brandon, M., Easley, K., Chen, E., Brown, M. D., Sukernik, R. I., Olckers, A., & Wallace, D. C. (2003). Natural selection shaped regional mtDNA variation in humans. Proceedings of the National Academy of Sciences, 100(1), 171–176. 10.1073/pnas.0136972100 PMC140917 12509511

[phy215049-bib-0028] Montero, D., Madsen, K., Meinild‐Lundby, A. K., Edin, F., & Lundby, C. (2018). Sexual dimorphism of substrate utilization: Differences in skeletal muscle mitochondrial volume density and function. Experimental Physiology, 103(6), 851–859. 10.1113/EP087007 29626373

[phy215049-bib-0029] Munguía‐Miranda, C., Sánchez‐Barrera, R. G., Tuz, K., Alonso‐García, A. L., & Cruz, M. (2009). Impaired fasting glucose detection in blood donors population. Revista Medica Del Instituto Mexicano Del Seguro Social, 47(1), 17–24. 19624959

[phy215049-bib-0030] Nielsen, J., & Christensen, D. L. (2011). Glucose intolerance in the West African Diaspora: A skeletal muscle fibre type distribution hypothesis. Acta Physiologica, 202(4), 605–616. 10.1111/j.1748-1716.2011.02272.x 21382179

[phy215049-bib-0031] O'Brien, T. R., Flanders, W. D., Decoufle, P., Boyle, C. A., DeStefano, F., & Teutsch, S. (1989). Are racial differences in the prevalence of diabetes in adults explained by differences in obesity? JAMA: the Journal of the American Medical Association, 262(11), 1485–1488. 10.1001/jama.1989.03430110075031 2769899

[phy215049-bib-0032] Paula, F. J., Pimenta, W. P., Saad, M. J., Paccola, G. M., Piccinato, C. E., & Foss, M. C. (1990). Sex‐related differences in peripheral glucose metabolism in normal subjects. Diabete Et Metabolisme, 16(3), 234–239. 2210019

[phy215049-bib-0033] Perry, C. G., Kane, D. A., Lin, C. T., Kozy, R., Cathey, B. L., Lark, D. S., Kane, C. L., Brophy, P. M., Gavin, T. P., Anderson, E. J., & Neufer, P. D. (2011). Inhibiting myosin‐ATPase reveals a dynamic range of mitochondrial respiratory control in skeletal muscle. Biochemical Journal, 437(2), 215–222. 10.1042/bj20110366 PMC3863643 21554250

[phy215049-bib-0034] Pesta, D., & Gnaiger, E. (2012). High‐resolution respirometry: OXPHOS protocols for human cells and permeabilized fibers from small biopsies of human muscle. Methods in Molecular Biology (Clifton, NJ), 810, 25–58. 10.1007/978-1-61779-382-0_3 22057559

[phy215049-bib-0035] Petersen, K. F., Befroy, D., Dufour, S., Dziura, J., Ariyan, C., Rothman, D. L., DiPietro, L., Cline, G. W., & Shulman, G. I. (2003). Mitochondrial dysfunction in the elderly: Possible role in insulin resistance. Science, 300(5622), 1140–1142. 10.1126/science.1082889 12750520 PMC3004429

[phy215049-bib-0036] Privette, J. D., Hickner, R. C., Macdonald, K. G., Pories, W. J., & Barakat, H. A. (2003). Fatty acid oxidation by skeletal muscle homogenates from morbidly obese black and white American women. Metabolism, 52(6), 735–738. 10.1016/S0026-0495(03)00034-9 12800100

[phy215049-bib-0037] Short, K. R., Bigelow, M. L., Kahl, J., Singh, R., Coenen‐Schimke, J., Raghavakaimal, S., & Nair, K. S. (2005). Decline in skeletal muscle mitochondrial function with aging in humans. Proceedings of the National Academy of Sciences, 102(15), 5618–5623. 10.1073/pnas.0501559102 PMC556267 15800038

[phy215049-bib-0038] Stuart, C. A., McCurry, M. P., Marino, A., South, M. A., Howell, M. E., Layne, A. S., Ramsey, M. W., & Stone, M. H. (2013). Slow‐twitch fiber proportion in skeletal muscle correlates with insulin responsiveness. The Journal of Clinical Endocrinology & Metabolism, 98(5), 2027–2036. 10.1210/jc.2012-3876 23515448 PMC3644602

[phy215049-bib-0039] Tanner, C. J., Barakat, H. A., Dohm, G. L., Pories, W. J., MacDonald, K. G., Cunningham, P. R., Swanson, M. S., & Houmard, J. A. (2002). Muscle fiber type is associated with obesity and weight loss. American Journal of Physiology‐Endocrinology and Metabolism, 282(6), E1191–E1196. 10.1152/ajpendo.00416.2001 12006347

[phy215049-bib-0040] Tarnopolsky, M. A., Rennie, C. D., Robertshaw, H. A., Fedak‐Tarnopolsky, S. N., Devries, M. C., & Hamadeh, M. J. (2007). Influence of endurance exercise training and sex on intramyocellular lipid and mitochondrial ultrastructure, substrate use, and mitochondrial enzyme activity. American Journal of Physiology‐Regulatory, Integrative and Comparative Physiology, 292(3), R1271–R1278. 10.1152/ajpregu.00472.2006 17095651

[phy215049-bib-0041] Toledo, F. G. S., Dubé, J. J., Goodpaster, B. H., Stefanovic‐Racic, M., Coen, P. M., & DeLany, J. P. (2018). Mitochondrial respiration is associated with lower energy expenditure and lower aerobic capacity in African American Women. Obesity (Silver Spring), 26(5), 903–909. 10.1002/oby.22163 29687648 PMC5918421

[phy215049-bib-0042] Toledo, F. G., Menshikova, E. V., Azuma, K., Radiková, Z., Kelley, C. A., Ritov, V. B., & Kelley, D. E. (2008). Mitochondrial capacity in skeletal muscle is not stimulated by weight loss despite increases in insulin action and decreases in intramyocellular lipid content. Diabetes, 57(4), 987–994. 10.2337/db07-1429 18252894

[phy215049-bib-0043] Warren, J. L., Bulur, S., Ovalle, F., Windham, S. T., Gower, B. A., & Fisher, G. (2017). Effects of acute hyperinsulinemia on skeletal muscle mitochondrial function, reactive oxygen species production, and metabolism in premenopausal women. Metabolism, 77, 1–12. 10.1016/j.metabol.2017.08.004 29132536 PMC5726454

[phy215049-bib-0044] Weston, A. R., Mbambo, Z., & Myburgh, K. H. (2000). Running economy of African and Caucasian distance runners. Medicine & Science in Sports & Exercise, 32(6), 1130–1134. 10.1097/00005768-200006000-00015 10862541

[phy215049-bib-0045] Wild, S., Roglic, G., Green, A., Sicree, R., & King, H. (2004). Global prevalence of diabetes: Estimates for the year 2000 and projections for 2030. Diabetes Care, 27(5), 1047–1053. 10.2337/diacare.27.5.1047 15111519

